# Massive facial keloid precipitated by chronic suppuration and mechanical skin traction: A case report

**DOI:** 10.1002/ski2.387

**Published:** 2024-04-11

**Authors:** Chihena H. Banda, Owen Ngalamika, Victor M. Zuze, Alick Bwanga, Chibamba Mumba

**Affiliations:** ^1^ Plastic and Reconstructive Surgery Unit Department of Surgery University Teaching Hospital Lusaka Zambia; ^2^ Department of Dermatology University Teaching Hospital Lusaka Zambia; ^3^ Department of Pathology and Microbiology University Teaching Hospital Lusaka Zambia; ^4^ Department of Surgery University Teaching Hospital Lusaka Zambia

## Abstract

Keloids are benign fibroproliferative tumours with a high recurrence rate of 20%–100%, therefore, multimodal treatment is recommended. We report the case of an exceptionally large facial keloid precipitated by a vicious cycle of chronic inflammation and mechanical skin traction and discuss the management challenges in a low resource setting. A 67‐year‐old man presented with a 10‐year history of a facial keloid that rapidly enlarged to 2,800 g in 2 years causing difficulties eating, speaking, dressing, head movements and breathing. He had multiple other smaller keloids, hypertension, HIV, and a keloid family history. Surgical excision of the keloid including the multiple sinuses and cysts of enclosed skin with growing hair found inside was performed. A posterior skin flap was used to achieve tension free closure and monthly triamcinolone injections commenced. Histology showed keloidal collagen bundles in a fibrotic background, foci of a lymphoplasmacytic infiltrate and multinucleated foreign body type giant cells consistent with chronic inflammation. CD34 and S100 immunohistochemistry were both negative, ruling out the differential diagnoses that included dermatofibroma. Recovery was uneventful and the patient was discharged after 2 weeks. Notably, radiotherapy was not available in our country. We report this unique case of an extremely large keloid to demonstrate the role of suppurative chronic inflammation and high skin tension in accelerated keloid growth. This case also highlights the severe global disparity in the availability of effective keloid treatment and the urgent need for access to radiotherapy services especially in Africa where keloid prevalence is highest.

## Graphical abstract

Keloids are fibroproliferative tumours strongly driven by genetics. They are most common in people of African descent and have a high recurrence rate, therefore multimodal treatment is recommended. We report an exceptionally large facial keloid precipitated by a vicious cycle of chronic inflammation and mechanical skin traction and discuss the management challenges. This case also highlights the severe global disparity in the availability of effective keloid treatment and the urgent need for radiotherapy services in Africa where keloid prevalence is highest.

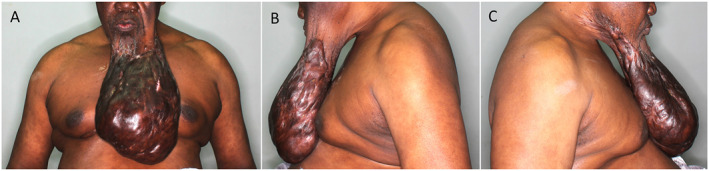

## 1 INTRODUCTION

Keloids are benign fibroproliferative tumours characterised by excessive accumulation of extracellular matrix components in the dermis and subcutaneous tissue. Symptoms include itching, pain, functional and cosmetic deformities. Keloids have a high recurrence rate of 20%–100%, therefore, multimodal treatment is recommended.^1^

We report the case of an exceptionally large facial keloid precipitated by a vicious cycle of chronic inflammation and mechanical skin traction of the enlarging keloid. We discuss the management challenges and highlight the growing global disparity in the availability of keloid care.

## 2 CASE REPORT

**FIGURE 1:**
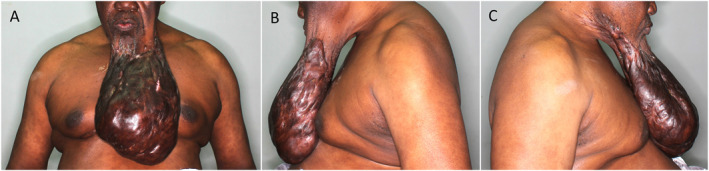
Pre‐operative photograph of the massive facial keloid on the lower jaw measuring 28 × 19.5 × 13 cm and weighing 2800 g. (1a) Shows the front, (1b) the left and (1c) the right side view. The patient had difficulty eating, speaking, wearing clothing, moving his head and sleeping in supine position due to the extremely large size and weight of the keloid.

A 67‐year‐old man was referred to our department from a rural hospital located 195 km away for treatment of a facial keloid. He presented with a 10‐year history of a progressively growing facial swelling associated with itchiness, pain, and periodic pus discharge from multiple sinuses (Figure 1). The keloid growth rapidly accelerated in the later 2 years with its extremely large size and weight causing difficulties eating, speaking, wearing clothing and with head and neck movement. The weight of the keloid also caused difficulties breathing when supine resulting in the patient resorting to sleeping on his side for relief. He had additional smaller keloids on his neck, chest and back, hypertension, family history of keloids in first degree relatives and was on antiretroviral therapy for HIV infection.

Pre‐operatively he was managed with oral cloxacillin, metronidazole and daily cleaning with antiseptic solution for 1 month while awaiting pre‐operative laboratory and imaging results. The pre‐operative CD4 count was 440 cells/μL. His blood electrolytes, kidney and liver function tests and liver enzymes were normal except for a raised lactate dehydrogenase of 328 IU/L. No special imaging of the keloid was performed.

**FIGURE 2:**
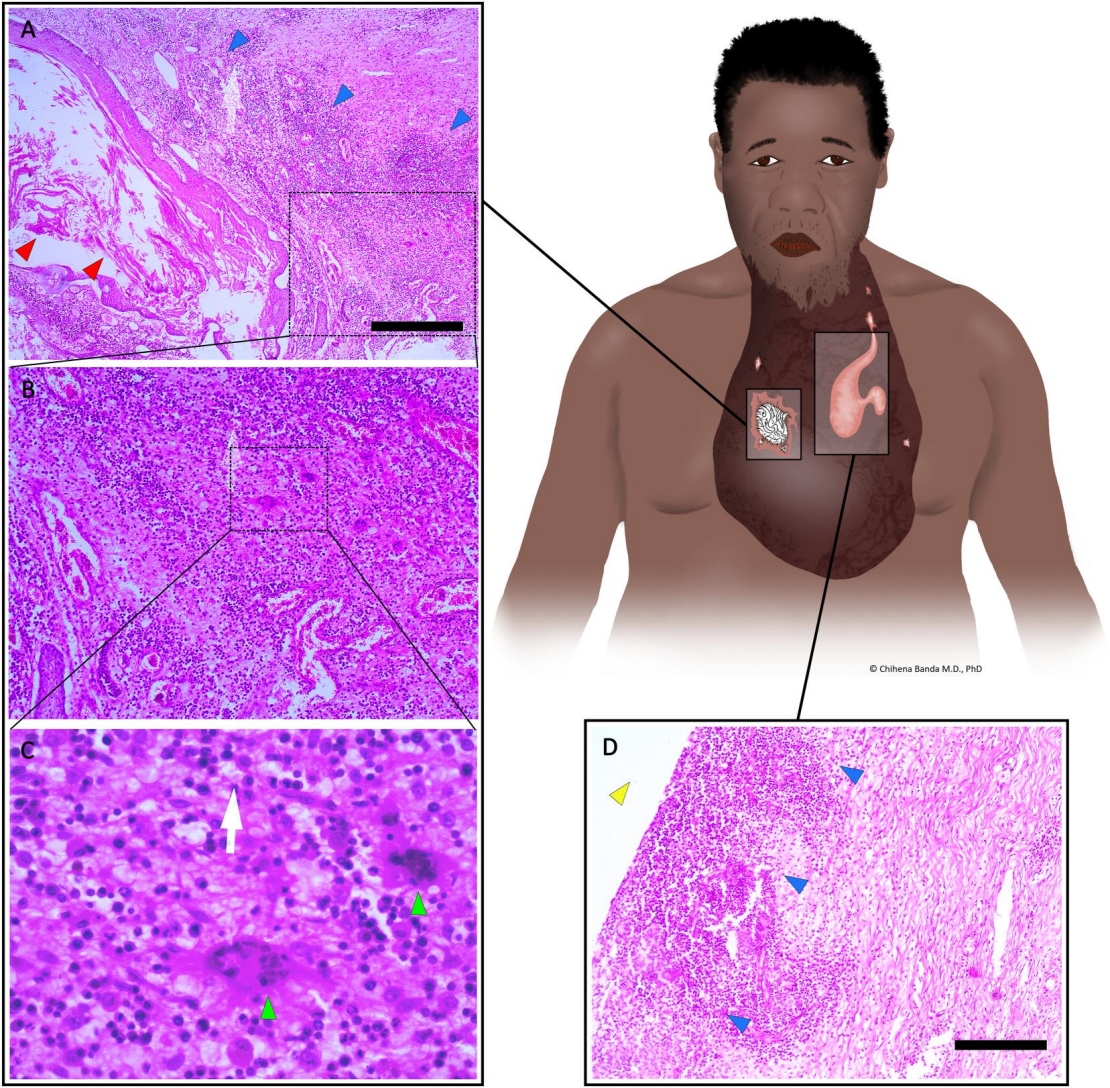
Histopathology following excision of the massive facial keloid stained with hematoxylin and eosin demonstrated benign surface stratified squamous epithelium and a subepithelial stroma with foci of keloidal collagen bundles in a background of fibrosis. The keloid contained multiple pus sinuses and multiple cysts of enclosed skin with tufts of long growing hair inside. 2a–c Shows the enclosed cystic lesions inside the keloid with stratified squamous epithelial lining and central flakes of keratin (red arrowhead) consistent with degrading hair follicles. The adjacent stroma demonstrated lymphocytic infiltrate (blue arrowheads) (2a) and multinucleate giant cells (green arrowheads) in a background of a mixed inflammatory infiltrate comprising mostly lymphocytes characteristic of chronic inflammation and foreign body reaction (2b and 2c). The pus sinuses showed foci of acute inflammation and suppurative inflammation with neutrophilic infiltrate (blue arrowheads) on the walls of the pus sinus cavities (yellow arrowheads) (2d). Immunohistochemistry for CD34 and S100 were both negative, ruling out a differential diagnosis of dermatofibroma. Scale bars = 500 μm.

Intra operatively, multiple sinuses were found with the largest extending to the keloid core and multiple cysts of enclosed skin with tufts of growing hair also found inside (Figure 2). The keloid weighing 2,800 g along with the sinuses and cysts was excised with a 10 × 7 cm posterior skin flap including part of the keloid wall epidermal and subepidermal layers used to achieve a tension free 2‐layered closure. A 16 F suction drain was placed under the flap and Triamcinolone 40 mg injected into the wound.

**FIGURE 3:**
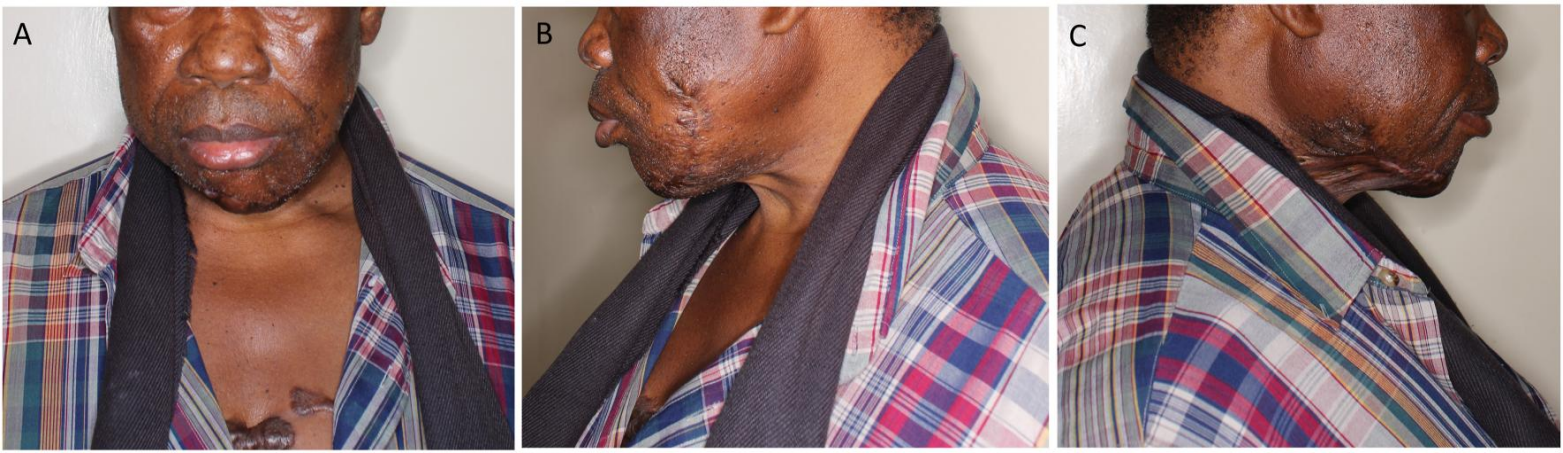
Post‐operative photograph of the patient 4‐month post excision of the massive facial keloid. (3a) Shows the front, (3b) the left and (3c) the right side view. Triamcinolone 40 mg was injected intra‐operatively immediately after wound closure and repeated monthly for 4 months. He did not receive any adjuvant radiotherapy as there was no available functional radiotherapy equipment in the country.

Recovery was uneventful with no complications. He was discharged 2 weeks post‐operation and triamcinolone injections repeated monthly for a planned 4 months (Figure 3). Radiotherapy was not available in the country. Histology demonstrated benign surface stratified squamous epithelium and a subepithelial stroma with foci of keloidal collagen bundles in a background of fibrosis. Cystic squamous epithelial islands whose walls demonstrated a chronic inflammatory infiltrate comprising lymphocytes, plasma cells and multinucleated giant cells were also seen (Figure 2). A focus of a neutrophilic infiltrate and suppurative inflammation was also seen. Immunohistochemistry for CD34 and S100 were both negative, ruling out a differential diagnosis of dermatofibroma.

## 3 DISCUSSION

Keloids are the result of chronic inflammation of the reticular dermis that causes sustained release of cytokines and growth factors, which in turn stimulate fibroblasts to proliferate and deposit excessive extracellular matrix components.^1^, ^2^ As such conditions that cause prolonged inflammation of skin wounds such as delayed closure, infections and foreign bodies increase the risk of keloid development.

In our patient the recurrent infection caused by multiple cysts and pseudo‐cysts/pus sinuses, and enclosed hair bearing skin resulted in a syndrome of recurrent pus discharge from the keloid known as suppurative keloid (SK). A recent study by Delaleu et al found SKs occur in up to 26% of keloid patients and identified several associated factors including male gender, African ancestry, pruritus, a folliculitis trigger, localization on the head, neck and limbs, and a positive keloid family history.^3^ They hypothesised that SKs were caused by fibrotic occlusion of pilosebaceous ostia (which are more frequent in the beard area) followed by cyst formation, inflammation, and liquefaction of the pilosebaceous contents and their eventual discharge through sinuses with the subsequent pilosebaceous unit's sebum rupture further causing a foreign body reaction, precipitating keloid growth.^3^ Both the clinical intra‐operative macroscopic findings and the histologic findings in our patient strongly support this hypothesis. Interestingly, the role of HIV infection in keloid progression is poorly understood.^4^ Whilst poor wound healing and the increased wound infections characteristic of HIV both increase the risk of keloid development, the immune dysfunction likely inhibits keloid progression, underscoring the complexity of the interaction between the two diseases and the need for further study.^2^

Mechanical skin tension has also been demonstrated to significantly contribute to keloid development and progression with a strong correlation observed between areas of high skin tension and keloid occurrence.^5^ Skin tension directly affects keloid growth through the process of mechanotransduction with the main mechanosignaling pathways including transforming growth factor *β*/Smad, integrin, mitogen‐activated protein kinase and G‐protein, tumor necrosis factor α/nuclear factor‐кB, Wnt/*β*‐catenin, interleukin, and calcium ion pathways.^5^ In our patient, both the severe mechanical skin tension caused by the sheer massive weight of the keloid hanging on the lower jaw, and the recurrent inflammation caused by the suppurative process jointly contributed to the rapid growth in a vicious cycle resulting in the massive keloid observed. Therefore, our treatment goal was to relieve the functional and aesthetic morbidity of the keloid by surgically addressing these two key drivers. In addition to triamcinolone, we would have preferred to administer adjuvant radiotherapy to reduce the risk of recurrence but this was unavailable.^6^ Radiotherapy has consistently been demonstrated to effectively reduce keloid recurrence and has been a major component of keloid treatment for over a century.^7^ Following the breakdown of the single radiotherapy device in Zambia, there has been no functioning radiotherapy unit in the country for the last 2 years^8^ During this period, patients have had to travel abroad for radiotherapy. This problem has disproportionately affected the poor from rural areas, such as our patient, who simply cannot afford the extremely high costs of treatment abroad resulting in many neglected patients developing extremely large and disabling keloids.

Sadly, this situation is not unique to Zambia with many other countries in Africa sharing this severe shortage of radiotherapy equipment.^9^ An updated analysis in 2021 found that no African country had a radiotherapy capacity that matched the estimated need, with 26 out of 54 countries having completely no radiotherapy.^10^ Keloids are among the commonest diseases treated by dermatologists and plastic surgeons worldwide, especially in sub‐Saharan Africa. Multiple studies have shown that keloids are 7–10 times more common in Blacks than Whites.^4^, ^11^ Furthermore, the reported keloid prevalence is as high as 10%–16% in sub‐Saharan Africa compared to 0.1%–1% in Asians and <0.1% in Europeans/North Americans.^11^, ^12^, ^13^, ^14^, ^15^ Thus this insufficiency of radiotherapy has left the population with the highest risk of keloids with no access to effective treatment. This largely ignored yet growing disparity in the availability of radiotherapy between high income countries and low‐middle income countries (LMIC) as highlighted in this case calls for urgent advocacy and concerted global action particularly towards dedicated financing for purchase and maintenance of radiotherapy equipment.

## 4 CONCLUSION

We report this unique case of an extremely large keloid to demonstrate the role of suppurative chronic inflammation and high skin tension in accelerated keloid growth. This case also highlights the severe global disparity in the availability of effective keloid treatment and the need for improved radiotherapy services in LMIC.

## FUNDING INFORMATION

This research received no specific grant from any funding agency in the public, commercial, or not‐for‐profit sectors.

## CONFLICT OF INTEREST STATEMENT

None to declare.

## AUTHOR CONTRIBUTIONS

**Chihena H. Banda**: Conceptualization (equal); investigation (equal); methodology (equal); supervision (equal); writing – original draft (equal); writing – review & editing (equal). **Owen Ngalamika**: Writing – review & editing (equal). **Victor M. Zuze**: Data curation (equal); investigation (equal); writing – original draft (equal); writing – review & editing (equal). **Alick Bwanga**: Investigation (equal); writing – review & editing (equal). **Chibamba Mumba**: Data curation (equal); investigation (equal); methodology (equal); writing – original draft (equal); writing – review & editing (equal).

## ETHICS STATEMENT

Approval for publication of this case report was obtained from the National Health Research Authority (ID: NHRA‐0001/31/01/2024). Full informed consent was obtained in writing from the patient to write and publish the case report including the images.

## Data Availability

The data underlying this article will be shared on reasonable request to the corresponding author. The study data is available on request from the authors.
